# The ManageHF Just-in-Time Adaptive Mobile App Interventions to Promote Self-Management and Improve Outcomes in Heart Failure: Randomized Controlled Trial

**DOI:** 10.2196/74121

**Published:** 2026-07-28

**Authors:** Michael P Dorsch, Mohamed S Ali, Amy Krambrink, Giselle Kolenic, Sabah Ganai, Juan Arzac, Xutong Zhang, Kaitlyn M Greer, Amit J Shah, Jennifer A Cowger, Gregory Ewald, Jo Ellen Rodgers, Dave L Dixon, Todd M Koelling, Scott L Hummel

**Affiliations:** 1 College of Pharmacy, University of Michigan–Ann Arbor, 248 Church Street, Ann Arbor, 48109, United States, 1 734-647-1452; 2 Frankel Cardiovascular Center, University of Michigan, Ann Arbor, MI, United States; 3 Biostatistics, School of Public Health, University of Michigan–Ann Arbor, Ann Arbor, MI, United States; 4 Department of Epidemiology, Rollins School of Public Health, Emory Univeristy, Atlanta, GA, United States; 5 Henry Ford Health System, Detroit, MI, United States; 6 School of Medicine, Washington University, St Louis, MO, United States; 7 Eshelman School of Pharmacy, University of North Carolina at Chapel Hill, Chapel Hill, NC, United States; 8 School of Pharmacy, Virginia Commonwealth University, Richmond, VA, United States; 9 Medical School, University of Michigan, Ann Arbor, MI, United States; 10 VA Ann Arbor Health System, Ann Arbor, MI, United States

**Keywords:** mobile health, mHealth, just-in-time adaptive interventions, self-management, heart failure, dietary sodium

## Abstract

**Background:**

Heart failure (HF) is a major health care challenge in the United States, with approximately 900,000 older adults hospitalized annually. Gaps in self-management, including unrecognized worsening symptoms and failure to adhere to dietary sodium restriction, can reduce quality of life and precipitate hospital admissions. Existing mobile health approaches to HF self-management have produced mixed results, highlighting the need for innovative strategies to improve postdischarge outcomes in at-risk patients.

**Objective:**

The ManageHF trial aimed to evaluate the effectiveness of 2 just-in-time adaptive interventions delivered via a mobile app to enhance HF self-management. The interventions focused on symptom recognition and lower dietary sodium restriction, with the goal of reducing readmissions and improving health-related quality of life (HRQOL) over a 12-week period.

**Methods:**

The trial was a 2×2 factorial, double-blind, randomized controlled study conducted across several US institutions. Participants recently hospitalized for acute HF were randomized into 4 groups: both interventions, either intervention alone, or an active control. The primary outcome was a composite measure assessing time to all-cause death, time to first HF readmission, and HRQOL changes, using the Minnesota Living with HF Questionnaire.

**Results:**

Recruitment was hindered by the COVID-19 pandemic, leading to the early discontinuation of the trial. Of the 62 participants enrolled, 43 completed the study. Participants were diverse, with a mean age of 55 (SD 14.4) years, 32% (20/62) were female, and 55% (34/62) identified as Black or African American. Most had HF with reduced ejection fraction. However, due to the early termination and small sample size, the ability to detect statistically significant differences was limited.

**Conclusions:**

The ManageHF trial highlighted the potential of mobile health technology to support HF management, particularly in enhancing HRQOL. Future studies using more effective recruitment and retention strategies are crucial for establishing the efficacy of these interventions with greater certainty.

## Introduction

Heart failure (HF) is the leading hospital discharge diagnosis among older adults in the United States, affecting 1.2 million patients in 2021 [1]. Roughly 20% of these patients are readmitted within 30 days, and half are readmitted within 6 months [2-8]. HF readmissions represent significant and potentially avoidable costs, with hospitalizations accounting for nearly 70% of annual HF costs [4]. Approximately one-third of patients who survive an HF hospitalization die within a year [9]. Many US hospitals have established quality improvement teams focused on 4 key domains: enhancing patient education, increasing the use of evidence-based therapies, ensuring timely postdischarge follow-up, and improving communication between inpatient and outpatient care providers [10,11]. However, despite these initiatives, results have been mixed, with recent improvements in readmission rates possibly attributable to changes in risk coding rather than actual outcomes [12,13]. Both crude and risk-adjusted 30-day mortality rates for HF have increased, indicating a need for new approaches to improve postdischarge outcomes [14,15].

Challenges with HF self-management, including failure to recognize clinical worsening and nonadherence to dietary sodium restrictions [2-6], are primary drivers of readmissions. Studies suggested that better dietary adherence, early help-seeking, and adequate medical support could prevent 23% to 31% of readmissions [3], reducing the 30-day readmission rate from approximately 20% to roughly 13.8% to 15.4%.

Self-monitoring has been shown to improve health-related quality of life (HRQOL) in patients with HF [16,17]. In a prospective single-center study, a web application designed to promote self-regulation was tested over 12 weeks, yielding positive outcomes for chronic HF symptoms [18]. Participants, predominantly older adults with New York Heart Association class II or III symptoms, used the application daily. Improvements were observed in the New York Heart Association classification and the Minnesota Living with HF Questionnaire (MLHFQ) scores, with notable numerical improvements in weight, exercise frequency, walking distance, jugular venous distension, and peripheral edema.

Dietary nonadherence, particularly excess dietary sodium intake, is a frequent precursor to HF decompensation and hospitalization [2-6]. Chronic neurohormonal activation and reduced renal perfusion in patients with HF promote sodium and fluid retention. Historically, guidelines have advocated a dietary sodium restriction of 2000 to 3000 mg/d [19,20], and current HF guidelines recommend avoiding “excessive” sodium intake [21]. In the Study of Dietary Intervention under 100 mmol in HF trial, aggressive sodium restriction did not reduce clinical outcomes. Still, it was associated with improved HRQOL [22] in stable outpatients with HF. The baseline sodium intake in the Study of Dietary Intervention under 100 mmol in HF was approximately 30% less than in US patients hospitalized for HF [23], who could derive more significant benefits from low-sodium dietary interventions [24].

The ManageHF trial aimed to evaluate the effectiveness of 2 just-in-time adaptive interventions (JITAIs) within a mobile app. These interventions targeted the two leading causes of hospital readmissions: (1) failure to recognize clinical worsening and (2) dietary nonadherence to lower-sodium food options. The study assessed the impact of JITAIs directed at HF symptoms and diet on mortality, hospital readmissions, and HRQOL over 12 weeks, hypothesizing that each intervention would yield reductions in mortality and readmissions, as well as improvements in HRQOL, as measured by the MLHFQ.

## Methods

### General Design

ManageHF was a 2 × 2 factorial, double-blind, randomized, controlled, multicenter clinical trial to determine the efficacy of JITAIs using a mobile app that focused on self-management and dietary support, either together or separately, compared with a control. Participants and study personnel were blinded to participant group assignment throughout the study. These interventions build upon previous work done by the research team [18,25,26]. The University of Michigan was the lead site, along with investigators at Henry Ford Health System, Virginia Commonwealth University, University of North Carolina, Washington University, University of Alabama at Birmingham, and Emory University. Eligible participants were randomized in a 1:1:1:1 manner to 4 conditions and stratified by site, gender, and HF type (HF with reduced ejection fraction vs HF with preserved ejection fraction). The four conditions were (1) diet and clinical worsening interventions (both), (2) diet intervention only, (3) clinical worsening intervention only, and (4) active control (neither intervention). All enrolled participants were asked to self-monitor with the mobile app, which included a daily survey, a blood pressure cuff, a scale, and a wearable activity monitor (3 devices provided to participants by Withings). The study protocol was updated throughout the study to reflect changes in the study workflow and updates to participant eligibility criteria. A full description of all protocol changes can be found in Multimedia Appendix 1. Advarra acted as the single institutional review board and approved this study. The trial was registered at ClinicalTrials.gov (NCT04755816; March 19, 2021) and was funded by the National Institute on Aging (R01AG062582).

### Study Participants

To participate in the study, individuals had to provide informed consent and meet the following criteria: be aged 18 years or older, hospitalized or recently hospitalized (within 14 d) for acute or acute-on-chronic decompensated HF, and fulfill specific heart function requirements, either left ventricular ejection fraction less than or equal to 40% or left ventricular ejection fraction greater than 40% with elevated B-type natriuretic peptide or N-terminal pro-B-type natriuretic peptide levels adjusted for BMI. Additional criteria included having a personal physician for follow-up, a compatible smartphone for the ManageHF app, a valid email address, being fluent in English, and providing signed informed consent. Participants were excluded if there was a contraindication to recommending a sodium-restricted diet; planned intervention for primary valvular heart disease during the study period; cardiac resynchronization therapy; on dialysis; previous cardiac transplantation or implantation of a ventricular assistance device or implantation expected within 3 months after randomization; cardiac or noncardiac illness with expected survival less than 3 months based on clinical judgment; listed status 1, 2, or 3 for a heart transplant; discharged to a setting other than their home; requirement for chronic inotropic therapy; inability to use Withings devices (weight scale, blood pressure monitor, and watch) due to equipment limitations or contraindications; or were currently pregnant or intended to become pregnant during the study period.

Potential participants were identified from current HF admissions at the enrollment site. The study teams were responsible for identifying eligible participants using inclusion and exclusion criteria. A potential participant was approached by a study team member to obtain consent before enrollment. Once enrolled, participants completed baseline questionnaires and surveys, created accounts with both Withings and ManageHF, and downloaded the ManageHF app.

### Intervention

The University of Michigan developed a mobile app named ManageHF, which was studied in this clinical trial. The app featured 2 novel JITAIs activated based on randomization. The mobile app aimed to facilitate self-management and behavioral change by providing participants with real-time, contextual feedback and support. This approach aimed to leverage adaptive technologies and personalized interventions to improve participants’ health outcomes and dietary habits.

The clinical worsening intervention focused on enhancing self-monitoring and self-management skills [27]. It used a health status indicator that adapted to users’ reported symptoms and provided feedback. The health status indicator was presented in a speedometer-like graphic that ranges from green (good health) to red (poor health) based on the answers to daily app questionnaires. Participants received 2 push notifications per day if they were in the yellow zone (fair health) and 3 per day if they were in the red zone, until they returned to the green zone. These notifications are linked to in-app educational resources to promote self-management behaviors. Personalization involved a baseline survey adapted from the Self-Care in HF Index [28] that gauged the user’s confidence and past behaviors in managing symptoms. These data helped tailor the messages for each user, using their name, emojis, and appropriate times of day to engage them effectively.

The dietary sodium intervention was designed to help users make lower-sodium choices by sending contextual messages linked to tailored in-app content [26]. The intervention notified the user when they were in a grocery store or restaurant. The Universal Product Code (UPC) in grocery stores helps to view tailored dietary information from the store, helping them identify and select lower-sodium options. Personalization was based on a confidence survey and a sodium intake screener, which asked users to identify their high-sodium foods and lower-sodium alternatives. Push notifications were purposely framed to align with the user’s confidence and dietary habits.

Both JITAIs relied on NumberEight, an internet-based service that uses various sensors to predict the user’s location. The NumberEight algorithm ran on the device, and data from these sensors were anonymized to ensure participant privacy and stored securely when the app was active. The study staff did not have access to participants’ location data.

### Outcomes

The primary outcome was a hierarchical composite of time to all-cause death, time to first HF readmission, and change from baseline to week 12 in the MLHFQ using the win ratio [29]. Death from any cause occurring during the study period was reported by each site. HF readmission was defined as admission to an inpatient unit or a stay in the emergency department following discharge after the index hospitalization, resulting in at least a 12-hour stay [30]. Only admissions associated with events that occur on an emergency or unplanned basis were considered potential end points and adjudicated. An HF readmission must have clinical manifestation of new dyspnea, orthopnea, paroxysmal nocturnal dyspnea, edema, pulmonary basilar rales or crackles, jugular venous distension, renal hypoperfusion with no other apparent cause, or radiological evidence of acute HF, and additional increased treatment with either intravenous HF medications (diuretics, inotropes, and vasodilators) or mechanical or surgical intervention directed explicitly at the treatment of HF. Only readmissions during the study period were assessed.

The MLHFQ tool comprises 21 questions that assess patients’ perceptions of the effects of HF on their daily lives [31]. The questions produce a total score ranging from 0 to 105, with higher scores indicating poorer HRQOL. The MLHFQ was collected at baseline, 6 weeks, and 12 weeks. A score change of 5 or more was considered a clinically significant difference for the hierarchical composite end point.

Nutritional questionnaires were used to assess the participants’ nutritional status and sodium intake. The Food Frequency Questionnaire (FFQ), collected at baseline, 6 weeks, and 12 weeks, was used to measure sodium intake. The 110-item Block FFQ (NutritionQuest, LLC) was electronically self-administered to record commonly consumed foods and estimate nutrient and energy intake.

### Statistical Approach

The primary outcome was a composite of death, HF readmission, and change in quality of life using the win ratio method, which incorporates a hierarchy of clinical prioritization on event occurrence. The win ratio was chosen to prioritize outcomes in a patient-focused fashion using a composite end point. Following the Finkelstein and Schoenfeld unmatched pairs win ratio method [32], each participant receiving the intervention was compared to each who did not receive the intervention, resulting in pairwise comparisons [29,33]. Within each pairing, the participant with the longest survival time is counted as the “winner” of that pairing. The win ratio is the number of winners divided by the number of losers associated with the intervention. Ties were excluded. The estimate of the win ratio and its 95% CI were calculated using bootstrap methods. The test for intervention effect is a nonparametric rank sum test. A win ratio of 1 indicates that both comparison groups have equivalent outcomes in terms of events and quality of life. In contrast, a win ratio greater than 1 indicates that the intervention group has a favorable profile compared to the control group, while a ratio less than 1 indicates an unfavorable profile. Using the intention-to-treat approach, the primary outcome was compared between each intervention and control. Based on standard 2 × 2 factorial designs, the clinical worsening intervention included those randomized to both the interventions and the clinical worsening-only conditions. The dietary sodium intervention included those randomized to both interventions and the dietary sodium–only conditions. The control for each intervention was the 2 remaining groups of the 4 randomized conditions.

The win ratio for the hierarchical end-point was estimated from our pilot study, where the win ratio was 1.33 in favor of the intervention group [27], meaning that people who received the intervention would be 33% more likely to experience favorable outcomes compared to the group not given the intervention. Assuming 10% dropout, 500 randomized participants (250 per main intervention arm) would provide 80% power to show a win ratio of 1.30 (56.6% winners in the intervention group excluding ties), assuming a 2-sided 5% type I error rate [29,32,33]. After accounting for dropout, 225 participants per arm would produce 50,400 (225 × 224) permutations of paired comparisons. The same reasoning was used to determine the power and sample size necessary to analyze the dietary sodium intervention. We did not design the study for sufficient power to detect an interaction effect of both interventions relative to the control.

For secondary end-points, each intervention versus control was examined in terms of individual components of the composite outcome using survival methods for time-to-event outcomes and linear mixed-effects models or piecewise linear regression for longitudinal continuous outcomes (eg, MLHFQ). Days alive and out of hospital (DAOH) were analyzed using Poisson regression. SAS (Statistical Analysis System; version 9.4; SAS Institute Inc) was used for all statistical analyses. Data are also presented in mean and SD for continuous variables normally distributed and median and IQR for those not normally distributed. Categorical variables are presented as a percentage of the total in that group. For completeness, outcome variables are presented across all 4 randomized conditions.

### Ethical Considerations

Advarra acted as the single institutional review board and approved this study (Pro00046349). Participants signed an informed consent document before participation in the clinical trial. After the study was completed, the data were deidentified for analysis and eventual release into a repository. Participants were compensated with US $200 for their time spent completing study-related tasks, and all participants were allowed to keep the devices provided during enrollment in the study. The reporting of the trial follows the CONSORT-EHEALTH (Consolidated Standards of Reporting Trials of Electronic and Mobile Health Applications and Online Telehealth) guidelines (Checklist 1).

## Results

### Overview

**Figure 1.**
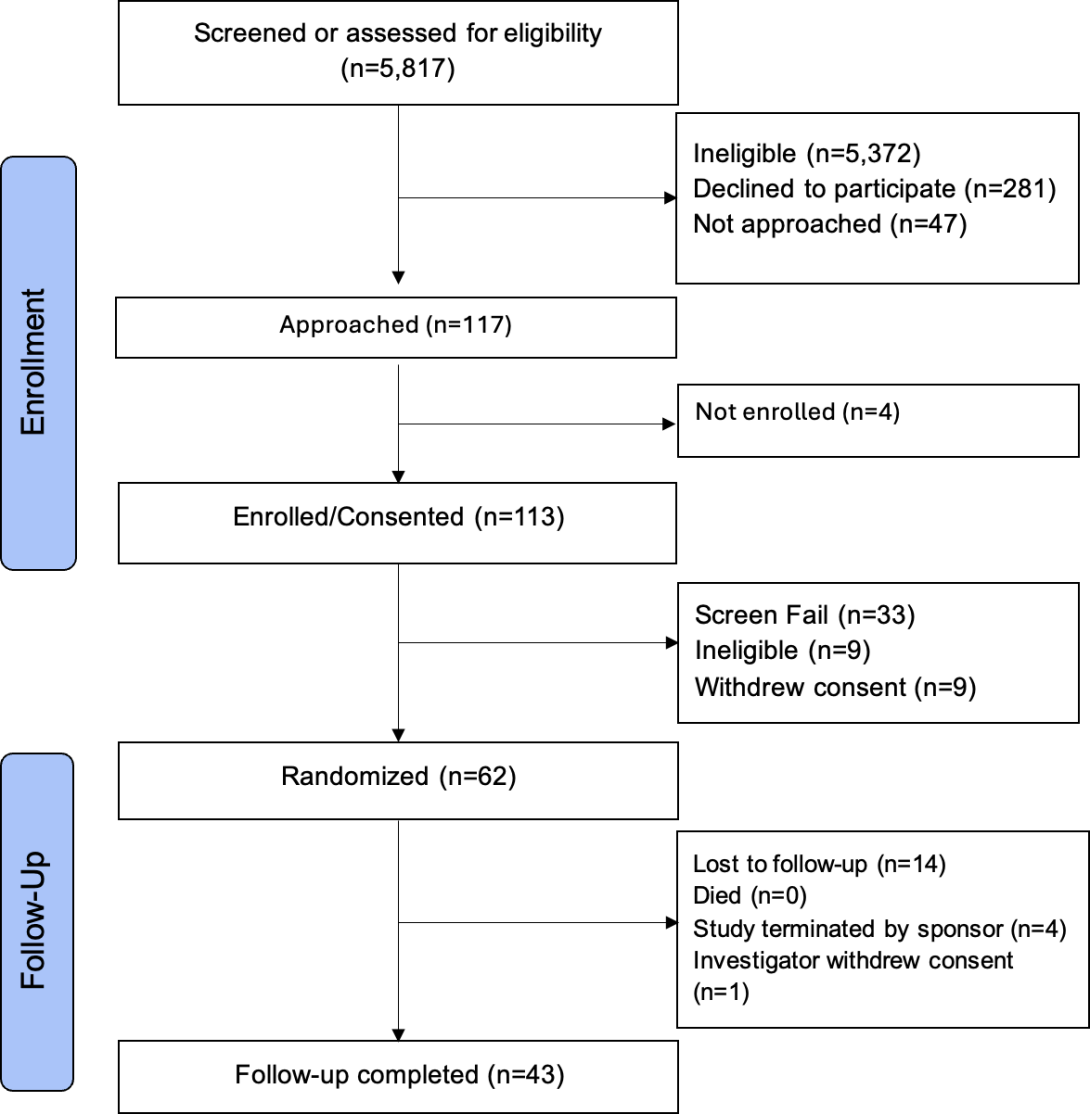
CONSORT (Consolidated Standards of Reporting Trials) diagram for the ManageHF trial.

The clinical trial was funded in April 2020, and enrollment began in June 2021. In February 2023, the Data and Safety Monitoring Board recommended discontinuing the clinical trial due to concerns regarding recruitment and retention. A total of 62 participants were randomized, but 43 completed follow-up. As requested by the funding agency, the National Institute on Aging, 19 participants were contacted in February 2023 to discontinue their participation in the study. This paper reports the trial’s results up until that point. Figure 1 shows the CONSORT (Consolidated Standards of Reporting Trials) diagram for this trial.

The mean age of participants was 55 (SD 14.4) years. Among the 62 participants, 32% (n=20) were female and 55% (n=34) identified as Black or African American. Most participants (n=48, 77%) were diagnosed with HF with reduced ejection fraction, and 40% (n=25) expressed class III symptoms. They had an average time from HF diagnosis of 2.9 (SD 4.56) years. Upon enrollment, 73% (n=45) were taking an aldosterone antagonist, and 48% (n=30) were taking an SGLT2 inhibitor. Table 1 demonstrates the baseline demographics overall and for each study group.

**Table 1.** Demographics of participants.

Characteristic or category	Overall (n=62)	Clinical worsening intervention group (n=30)	Clinical worsening control group (n=32)	Dietary sodium intervention group (n=38)	Dietary sodium control group (n=24)
Age (y), mean (SD)	54.8 (14.4)	54.5 (14.6)	55.1 (14.5)	54.4 (15.9)	55.4 (12.1)
Female, n (%)	20 (32)	10 (33)	10 (31)	13 (34)	7 (29)
Hispanic or Latino, n (%)	2 (3)	0 (0)	2 (6)	1 (3)	1 (4)
Race, n (%)					
Black or African American	34 (55)	14 (47)	20 (63)	20 (53)	14 (58)
White	25 (40)	15 (50)	10 (31)	16 (42)	9 (38)
Other or unknown	3 (5)	1 (3)	2 (6)	2 (5)	1 (4)
Ischemic etiology of HF^a^, n (%)	14 (23)	6 (20)	8 (25)	10 (26)	4 (17)
HF type, n (%)					
Reduced ejection fraction	48 (77)	22 (73)	26 (81)	28 (74)	20 (83)
Preserved ejection fraction	14 (23)	8 (27)	6 (19)	10 (26)	4 (17)
NYHA^b^ class, n (%)					
I	6 (10)	1 (3)	5 (16)	3 (8)	3 (13)
II	17 (27)	7 (23)	10 (31)	12 (32)	5 (21)
III	25 (40)	15 (50)	10 (31)	13 (34)	12 (50)
IV	3 (5)	2 (7)	1 (3)	3 (8)	0 (0)
Missing	11 (18)	5 (17)	6 (19)	7 (18)	4 (17)
Number of hospitalizations due to HF within the prior 12 months, n (%)					
0	20 (32)	11 (37)	9 (28)	15 (39)	5 (21)
1	22 (35)	10 (33)	12 (38)	11 (29)	11 (46)
2+	20 (32)	9 (30)	11 (34)	12 (32)	8 (33)
Previous MI^c^, n (%)	15 (24)	7 (23)	8 (25)	7 (18)	8 (33)
History of hypertension, n (%)	47 (76)	21 (70)	26 (81)	30 (79)	17 (71)
Serum creatinine, mean (SD)	1.4 (0.9)	1.3 (0.5)	1.5 (1.2)	1.5 (1.1)	1.3 (0.5)
Heart rate, mean (SD)	81.8 (15)	81.2 (14)	82.4 (15)	84.3 (12)	77.8 (18)
Systolic blood pressure, mean (SD)	119.5 (19)	115.6 (19)	123.2 (18)	118.1 (21)	121.7 (14)
BNP^d^					
n (%)	44 (71)	21 (70)	23 (72)	29 (76)	15 (63)
Mean (SD)	788.1 (846)	824.8 (640)	754.7 (1012)	738.7 (868)	883.8 (822)
NT-proBNP^e^					
n (%)	13 (21)	7 (23)	6 (19)	6 (16)	7 (29)
Mean (SD)	5562.3 (7402)	7006.7 (10040)	3877.2 (2044)	6937 (11092)	4384 (1877)
ACE^f^ or ARB^g^ or ARNI^h^, n (%)	45 (73)	23 (77)	22 (69)	27 (71)	18 (75)
Beta-blocker, n (%)	48 (77)	22 (73)	26 (81)	27 (71)	21 (88)
Aldosterone antagonist, n (%)	35 (56)	14 (47)	21 (66)	19 (50)	16 (67)
SGLT2i^i^, n (%)	30 (48)	15 (50)	15 (47)	21 (55)	9 (38)
MLHFQ^j^, mean (SD)	67.9 (23.65)	74.7 (20.56)	61.5 (24.85)	70.7 (21.43)	63.4 (26.65)
Sodium screener (mg/d), mean (SD)	3873.1 (1907)	4133.3 (2021)	3629.6 (1793)	3818.5 (2118)	3960.9 (1550)
Sodium FFQ^k^ (mg/d), mean (SD)	4064.9 (2642)	4170.9 (2758)	3962.4 (2567)	3998.7 (2760)	4176.1 (2489)

a HF: heart failure.

b NYHA: New York Heart Association.

c MI: myocardial infarction.

d BNP: B-type natriuretic peptide.

e NT-proBNP: N-Terminal pro-B-type natriuretic peptide.

f ACE: angiotensin-converting enzyme.

g ARB: angiotensin receptor blocker.

h ARNI: angiotensin receptor neprilysin inhibitor.

i SGLT2i: sodium-glucose cotransporter-2 inhibitor.

j MLHFQ: Minnesota Living with Heart Failure Questionnaire.

k FFQ: Food Frequency Questionnaire.

### Participant Engagement

Regardless of subgroup, all participants were asked to engage with the ManageHF app survey and use remote device monitoring on a daily basis for the 84-day study. Of the 62 participants enrolled in the clinical trial, 66.1% (41/62) completed at least one app survey. Among the 41 participants who completed at least 1 survey, the median was 22 (IQR 2-47) days. The wearable device was used by 78.5% (47/62) of participants. The median number of days using the device was 74 (IQR 39-84). Blood pressure was measured by 78.5% (47/62) of participants. Participants measured blood pressure on a median of 35 (IQR 15-51) days.

### Clinical Worsening Intervention

The hierarchical analysis of the overall primary end point showed a numerically larger number of wins in the clinical worsening intervention compared to the control group (346/960, 36% vs 200/960, 20.8%; win ratio 1.73, 95% CI 0.81‐4.44; *P*=.18). Both individual outcomes of time to HF readmission and change in MLHFQ demonstrated a larger number of wins in the clinical worsening intervention compared to the control group (HF readmissions: 201/960, 20.9% vs 109/960, 11.4%; MLHFQ: 145/960, 15.1% vs 91/960, 9.5%). Table 2 summarizes the hierarchical analysis of the primary end point in participants randomized to the clinical worsening intervention versus control. There were no deaths in the clinical trial. HF readmission was 13.3% (4/30) in the clinical worsening intervention and 21.8% (7/32) in the control group (hazard ratio 0.56, 95% CI 0.16-1.90; *P*=.35). From baseline to 12 weeks, the MLHFQ improved by a mean of 42 (SD 28) points in the clinical worsening intervention and by a mean of 29 (SD 28) points in the control group (*P*=.21). The mean number of DAOH was 83 (SD 22.45) in the clinical worsening group and 84 (SD 22.65) in the control group (*P*=.7).

Out of the 30 participants in the clinical worsening intervention group, 16 received 1066 push notifications during the study period, classified as yellow or red. Throughout the study, these participants received a median of 24 (IQR 6-70) push notifications.

**Table 2.** Hierarchical end point and win ratio for both interventions^a^^b^.

Outcome	Clinical worsening intervention wins, n (%)	Clinical worsening control wins, n (%)	Ties, n (%)	Dietary sodium intervention wins, n (%)	Dietary sodium control wins, n (%)	Ties, n (%)
Time to all-cause death	0 (0)	0 (0)	—^c^	0 (0)	0 (0)	—
Time to heart failure readmission	201 (20.9)	109 (11.4)	—	136 (14.9)	156 (17.1)	—
Change in MLHFQ^d^ from baseline to 12 week	145 (15.1)	91 (9.5)	—	147 (16.1)	61 (6.7)	—
Overall (across 3 outcomes)	346 (36)	200 (20.8)	414 (43.1)	283 (31)	217 (23.8)	412 (45.2)

a Clinical worsening intervention versus control: win ratio 1.73 (95% CI 0.81–4.44); *P*=.18.

b Dietary sodium intervention vs control: win ratio 1.30 (95% CI 0.57–3.26); *P*=.53.

c —: not applicable.

d MLHFQ: Minnesota Living with Heart Failure Questionnaire.

### Dietary Sodium Intervention

The hierarchical analysis of the primary end point, as shown in Table 2, demonstrated a numerically larger number of wins in the dietary sodium intervention compared with the control group (283/912, 31% vs 217/912, 23.8%; win ratio 1.30, 95% CI 0.57‐3.26; *P*=.53). The change in MLHFQ showed a larger number of wins in the dietary sodium intervention compared to the control group (147/912, 16.1% vs 61/912, 6.7%). From baseline to 12 weeks, the MLHFQ improved by 39 (SD 34) points in the dietary sodium intervention and by 24 (SD 25) points in the control group (*P*=.17). In contrast, the dietary sodium intervention group had a numerically smaller number of wins than the control group in terms of the outcome time to HF readmission (136/912, 14.9% vs 156/912, 17.1%). HF readmission was 18.4% (7/38) in the dietary sodium intervention and 16.7% (4/24) in the control group (hazard ratio 1.10, 95% CI 0.32-3.74; *P*=.88). By FFQ at 12 weeks, sodium intake was 2359 (SD 2770) mg in the intervention and 2142 (SD 1671) mg in the control group (n=32; *P*=.23). By sodium screener at 12 weeks, sodium intake was 2122 (SD 1280) mg in the intervention and 2390 (SD 796) mg in the control group (n=30; *P*=.89). The mean number of DAOH was 86 (SD 18.49) in the dietary sodium intervention and 82 (SD 21.96) in the control group (*P*=.16).

Of the 38 participants in the dietary intervention group, 23 received 786 notifications at grocery stores or restaurants. Throughout the study, these participants received a median of 10 (IQR 2-41) push notifications.

### Both Interventions

Across all 4 conditions, 18 participants were randomized to both interventions, 12 to the clinical worsening intervention only, 20 to the dietary sodium intervention only, and 12 to neither intervention. HF readmission was 11.1% (2/18) for those randomized to both interventions, 16.7% (2/12) in the clinical worsening only group, 25% (5/20) in the dietary sodium only group, and 16.7% (2/12) for those in neither intervention. The change in MLHFQ showed a 47 (SD 28) point improvement for those in both interventions, a 31 (SD 29) for those in the clinical worsening only group, a 34 (SD 38) point improvement in the dietary sodium only group, and a 19 (SD 22) point improvement for those assigned to neither intervention. There were no statistically significant differences between these 4 conditions.

## Discussion

This study was a novel trial of 2 JITAIs delivered via a mobile app, using push notifications and in-app content, at 2 critical time points in HF self-care: when a patient feels worse and when dietary sodium support is needed when entering grocery stores and restaurants. Although the primary end-point showed a trend in both intervention groups relative to control, it did not reach statistical significance and was substantially underpowered due to early stopping. The early discontinuation of the trial resulted in a smaller-than-planned sample size (62 participants, of whom only 43 completed follow-up). This limited the statistical power to detect significant differences between the intervention and control groups. The reduced number of participants limits the generalizability of the findings and is an important limitation of this study. This trial demonstrates innovative methods that, unfortunately, were implemented during a global pandemic that significantly hindered clinical research.

Several mobile app–based interventions for HF [34-36] and sodium intake [37-39] have been studied. While several of these studies implement self-monitoring to drive self-management in HF, none provide a JITAI using several factors to personalize the intervention for success. The ManageHF app provides self-care push notifications and in-app educational content when patients are experiencing worsening symptoms. The self-care push notifications were personalized to the self-care methods the participant had found effective in the past. A bank of over 50 push notifications paired with detailed in-app educational content was used for this intervention. Push notifications and in-app content did include information about guideline-directed medical therapy, medication adherence, and loop diuretic efficacy. The ManageHF app did not directly provide information about adding or titrating guideline-directed medical therapy. The lack of medication optimization is one of the most critical interventions in HF to reduce hospitalizations, and digital health interventions in this area are essential [40].

In a recent systematic review of mobile health interventions aimed at reducing sodium intake, only 6 were randomized controlled clinical trials [38]. Of those trials, only 2 were mobile app interventions published in English. One of those apps is SaltSwitch, which originates from New Zealand [37]. SaltSwitch was designed to help users locate food products with low sodium levels at grocery stores. However, the SaltSwitch app does not personalize recommendations based on the user’s specific requirements for high-sodium foods. Additionally, it lacks features such as push notifications to remind users to scan items and does not provide contextual food information for restaurants. SaltSwitch has not been studied in HF to date. Two quasi-experimental dietary intervention studies were conducted in patients with HF, and neither involved mobile apps. One was a game-based interactive tool for self-management [41], and another was an SMS text messaging intervention [42]. Neither study assessed clinical outcomes.

The National Institute on Aging funded this trial in April 2020, 1 month after the United States declared a nationwide public health emergency, and the same month, more than 1 million cases of COVID-19 were confirmed worldwide. Multiple funding agencies issued statements to researchers regarding collaboration during this challenging period to facilitate research [43,44]. Due to the COVID-19 pandemic, significant challenges arose in all research areas [45]. Many of our sites saw unprecedented issues with access to their health care facilities, and resources for research were impacted. All study sites experienced research closures, which were site-specific, meaning that at any given time, only some of our research sites were recruiting participants. There were also participant-level challenges in recruiting patients with HF. During the COVID-19 pandemic, HF hospitalizations declined significantly [45-47], resulting in [46-48] a smaller patient pool to recruit from and a lower-than-expected HF readmission rate for our clinical trial. Potential participants may have been less likely to enroll due to societal or individual pandemic-related circumstances. Additionally, participants who did enroll were less likely to visit restaurants and grocery stores during the pandemic, which is a limitation of this trial.

Demographic diversity is a crucial discussion point in clinical trials [49], particularly in those discontinued by the Data and Safety Monitoring Board due to low recruitment. Black participants represented 55% (34/62) of the enrolled population in this study, compared to less than 10% in other recent randomized trials enrolling patients with HF [50-53]. Recruiting and enrolling diverse patients in clinical trials is vital, but it requires additional staff resources and time. We have shown that enrolling diverse participants from a federally qualified health center required 1.4 times the full-time equivalent staff compared to a less diverse university hospital setting [54]. This is even more important in mobile health–related clinical trials, where interventions can exacerbate inequities [55].

An important aspect of this clinical trial is the inclusion of an active control. All participants received a blood pressure cuff, a scale, and a wearable activity monitor and were asked to self-monitor using the mobile app, which included a daily survey. This was done to fulfill the third aim of the research grant, which was to determine if passive preclinical measurements from these devices could predict symptoms of worsening HF. Using an active control can reduce the chance of detecting a statistically significant outcome. An active control can also add rigor to the trial because it biases the results toward showing no difference between the groups. This should be accounted for in the clinical trial statistical power assessment and is a limitation of this study.

Despite pandemic-related recruitment challenges, the ManageHF study successfully engaged a diverse cohort of high-risk patients with HF in self-management activities. Future studies using more effective recruitment and retention strategies are crucial for establishing the efficacy of these interventions with greater certainty.

## Supplementary material

10.2196/74121 Multimedia Appendix 1 Protocol changes or updates.

10.2196/74121 Checklist 1 CONSORT-EHEALTH checklist.

## Abbreviations

CONSORT: Consolidated Standards of Reporting Trials
CONSORT-EHEALTH: Consolidated Standards of Reporting Trials of Electronic and Mobile Health Applications and Online Telehealth
DAOH: days alive and out of hospital
FFQ: Food Frequency Questionnaire
HF: heart failure
HRQOL: health-related quality of life
JITAI: Just-in-time Adaptive Intervention
MLHFQ: Minnesota Living with Heart Failure Questionnaire
SAS: Statistical Analysis System
UPC: Universal Product Code
