# Supplementary material for: The ManageHF Just-in-Time Adaptive Mobile App Interventions to Promote Self-Management and Improve Outcomes in Heart Failure: Randomized Controlled Trial
Source: JMIR Mhealth Uhealth. 2026 Jul 28;14:e74121. doi: 10.2196/74121 (PMC13412010; doi:10.2196/74121)
Supplement: Multimedia Appendix 1 [file mhealth-v14-e74121-s001.pdf]

Version v1.1.2020.12.18:

Changes to streamline workflow and improve consistency in assessments were made. The timing of the HADS and nutritional assessments was moved from the day of discharge to the screening/enrollment phase, ensuring coordinators could assist participants and complete data collection efficiently. Additionally, HRQOL and nutritional assessments were added to the screening/baseline visit for alignment with the protocol's schedule of activities, enhancing internal consistency.

Version v1.2.2021.04.09:

Clarified the smartphone inclusion criterion to ensure participants have a compatible Apple or Android device capable of downloading and using the ManageHF app with all permissions accepted. This adjustment was made to guarantee the app functions as intended and supports seamless study participation.

Version v2.0.2021.12.03:

The study workflow was updated in sections 1.2, 5.1, and 5.2 to reflect new visit windows, allowing screening, recruitment, enrollment, and baseline visits up to 7 days post-discharge. Visit windows for weeks 6 and 12 were extended to +/-10 days, and the 1-question NHYA survey was removed as a randomization requirement. The process for randomizing re-hospitalized participants was clarified. Inclusion criteria were adjusted to remove redundant text and allow enrollment up to 7 days post-discharge. Exclusion criteria were updated to include low sodium intake and re-hospitalization prior to randomization. Adverse events of special interest (AESI) and serious adverse events (SAEs) reporting timelines were clarified based on hospitalization status. Additional changes included allowing paper copies for lengthy questionnaires and reordering baseline questionnaires for administrative consistency.

Version v3.0.2022.04.22:

The inclusion and exclusion criteria in sections 1.2, 5.1, and 5.2 were updated to address lower-than-expected recruitment by broadening the pool of eligible heart failure patients. The inclusion criteria changes include removing the left atrial size >40mm requirement, lowering BNP from 200 to 175 pg/ml, and NT-proBNP from 800 to 700 pg/ml, and adjusting thresholds for NT-proBNP and BNP based on BMI. The exclusion criteria now include percutaneous cardiac or carotid procedures, carotid stenting, TAVR within 1 month prior to screening, and major cardiovascular surgery within 3 months prior to screening. Other changes include replacing end-stage renal disease with dialysis, removing end-stage liver disease, adding similar devices to the exclusion of previous cardiac transplantation or ventricular assistance device implantation, listing heart transplant status 1, 2, or 3, and reducing non-cardiac illness expected survival from 6 months to 3 months. Compensation details were added, offering $50 at baseline, $50 at week 6, and $100 at week 12, along with texting as a contact method to improve recruitment and retention, as recommended by the DSMB.

Version v3.1.2022.09.27:

The inclusion and exclusion criteria were updated in sections 1.2, 1.3, 5.1, and 5.2 to address lower-than-expected recruitment by broadening the pool of eligible patients with heart failure. The inclusion criteria now allow for discharge dates up to 14 days post-discharge. Several exclusion criteria were removed, including acute coronary syndrome, stroke, transient ischemic attack, percutaneous cardiac or carotid procedure, major cardiovascular surgery within 3 months prior to screening, known active myocarditis, hypertrophic obstructive cardiomyopathy, pericarditis, cardiac amyloidosis, restrictive cardiomyopathy, complex congenital heart disease, severe stenotic heart disease, severe uncorrected thyroid disease, and home oxygen-dependent lung disease. These changes were approved by the DSMB to enhance recruitment efforts.
